# Point of Care Ultrasonography to Monitor Decongestive Therapy in Heart Failure: Seeing is Believing 

**DOI:** 10.24908/pocus.v7iKidney.15340

**Published:** 2022-02-01

**Authors:** Abhilash Koratala

**Affiliations:** 1 Division of Nephrology, Medical College of Wisconsin Wisconsin, Milwaukee USA

**Keywords:** VExUS, POCUS, point of care ultrasound, nephrology, heart failure, D-sign

## Case

Point of care ultrasonography (POCUS) is increasingly being used as an adjunct to physical examination in internal medicine and subspecialties. In nephrology practice, it is particularly valuable in the objective assessment of fluid volume status. In addition, POCUS enables the physicians to better appreciate the anatomic and pathophysiologic changes that occur during the course of patient management. The intent of this case file is to demonstrate the improvement in sonographic markers of congestive state during ultrafiltration therapy in a patient with heart failure.

**Figure 1:**
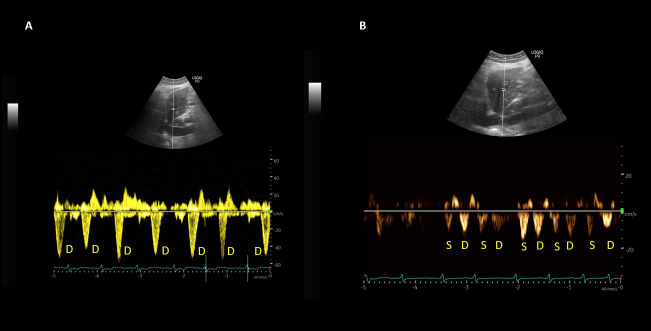
(A) Hepatic vein Doppler waveform at the initiation of ultrafiltration therapy demonstrating only diastolic (D) wave below the baseline indicating severe congestion. (B) Follow up waveform demonstrating both systolic (S) and diastolic (D) waves below the baseline, indicating improved venous return. Note the correlation with accompanying EKG.

**Figure 2:**
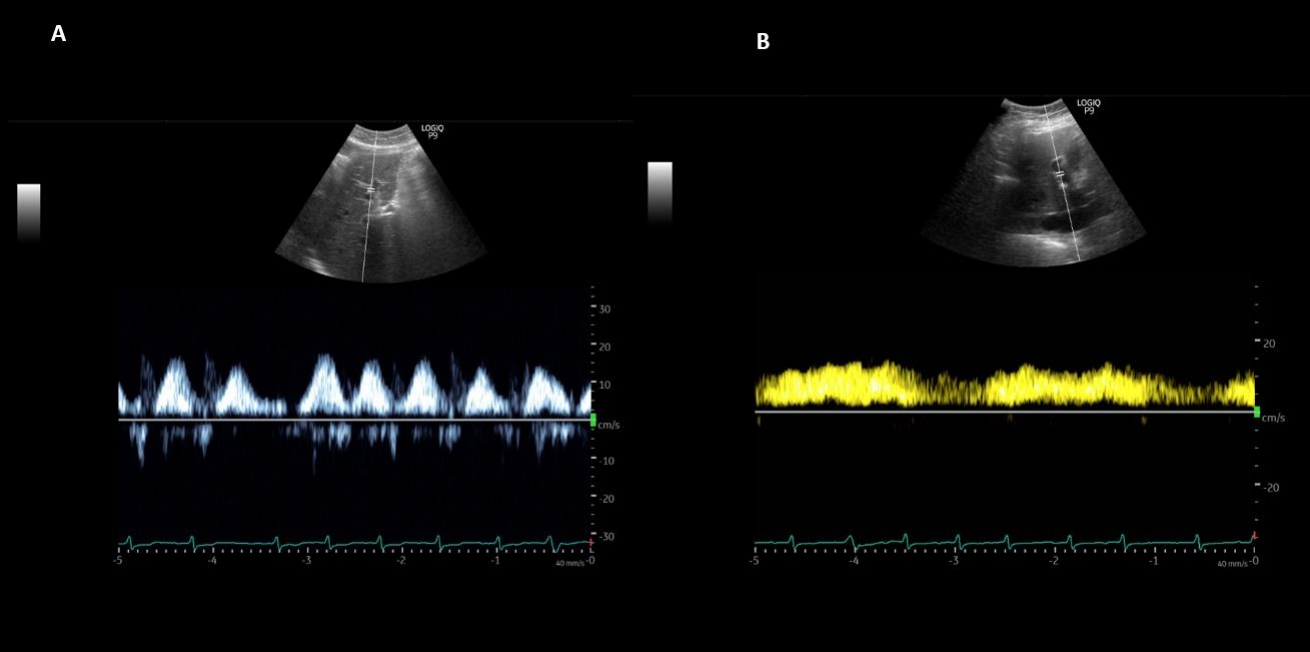
(A) Portal vein Doppler waveform at the initiation of ultrafiltration therapy demonstrating pulsatile pattern consistent with severe congestion. (B) Follow up waveform demonstrating considerable improvement in thepulsatility (almost normal, continuous pattern).

A 74-year-old gentleman with a history of hypertension, diabetes mellitus type 2, coronary artery disease, congestive heart failure with reduced left ventricular ejection fraction (<20%), and chronic kidney disease stage 3 was admitted to the hospital for acute exacerbation of heart failure. He presented with a serum creatinine of 2.7 mg/dL, which slowly trended down to 2.3 mg/dL with intravenous loop diuretic therapy over the next 5 days and started to trend up again prompting nephrology consultation. As the serum creatinine continued to rise (4.1 mg/dL) with oliguria and hypotension (systolic blood pressure 85-90 mmHg), continuous renal replacement therapy was initiated. He was also started on dobutamine drip by the cardiology team. A POCUS examination was performed by the nephrologist including a focused echocardiogram, lung ultrasound, and a venous excess Doppler ultrasound (VExUS). Echo was significant for a plethoric inferior vena cava (IVC) measuring approximately 2.5 cm in maximal diameter (online Video S1A) with almost no respiratory variation consistent with very high right atrial pressure. There was significantly decreased left ventricular ejection fraction. Parasternal short axis view demonstrated a ‘D’-shaped left ventricle with interventricular septal flattening throughout the cardiac cycle suggestive of right ventricular pressure and volume overload (online Video S1B). It was likely that the compression of the left ventricle was leading to a reduction in stroke volume, contributing to hypotension. Lung ultrasound demonstrated B-line pattern bilaterally, consistent with pulmonary edema/interstitial syndrome (online Video S1C). VExUS scan demonstrated D-only pattern on hepatic vein Doppler, which means there is flow towards the heart only during diastole (severe congestion, Figure 1A). Similarly, portal vein Doppler was suggestive of severe congestion with 100% pulsatility and possible flow reversal during systole (Figure 2A). Based on these findings, the rate of ultrafiltration was increased despite hypotension and the patient was intubated soon after this scan was performed. A repeat POCUS examination was performed 4 days later after approximately 10 liters net ultrafiltration. The patient remained intubated at that time though the systolic blood pressure improved to ~110 mmHg. Inferior vena cava was still plethoric (on mechanical ventilation, online Video S2A) but the venous Doppler showed significant improvement in hepatic and portal vein waveforms. Both S (systolic) and D (diastolic) waves were below the baseline on the hepatic vein tracing indicating near-normal venous return (Figure 1B) and the portal vein pulsatility has substantially improved to ~30% (Figure 2B). Interestingly, the left ventricle appeared round on the parasternal short axis view suggesting improvement in right ventricular volume and pressure overload and thus improved left ventricular outflow (online Video S2B). As expected, lung ultrasound showed improvement with predominantly A-line pattern though there were B-lines at the bases (online Video S2C). In addition, the urine output also picked up though the patient was never completely anuric.

In summary, it is important for the nephrologists to be aware that not all hypotension is due to volume depletion and cases of severe congestion with dilated right ventricle compromising the left ventricular outflow as demonstrated here will respond to volume removal 1, 2. In addition, flow patterns in hepatic and portal veins (renal parenchymal vein where feasible) can be used to monitor the efficacy of decongestive therapy^3^, ^4^, ^5^, ^6^ in real time, even in cases where the inferior vena cava remains enlarged due to chronic pulmonary hypertension and/or positive pressure ventilation. 

## Conflict of Interest

The author declares that no conflict of interest exists.

## Consent

Informed consent has been obtained from the patient for the publication of this case study.

## Supplementary Material

 Video S1 Sonographic parameters at the initiation of ultrafiltration therapy: (A) plethoric inferior vena cava, >2 cm in diameter; (B) D-sign on parasternal short axis view of the heart, demonstrating interventricular septal flattening due to increased right ventricular pressure making the left ventricle (LV) appear like the letter ‘D’; (C) Lung ultrasound demonstrating B-lines (representative image from right anterior scan zone).

Video S2 Sonographic parameters at follow up: (A) Inferior vena cava still plethoric (on mechanical ventilation); (B) parasternal short axis view of the heart demonstrating a round left ventricle (LV) and disappearance of the D-sign; (C) Lung ultrasound demonstrating A-lines (same scan zone as Video S1).
